# Predictive Power of Tissue and Circulating Biomarkers for the Severity of Biopsy-Validated Chronic Liver Diseases

**DOI:** 10.3390/jcm11205985

**Published:** 2022-10-11

**Authors:** Guido Bocci, Paola Orlandi, Maria Laura Manca, Chiara Rossi, Antonio Salvati, Maurizia Rossana Brunetto, Anna Solini

**Affiliations:** 1 Department of Clinical and Experimental Medicine, University of Pisa, Via Roma 55, 56126 Pisa, Italy; 2 Department of Mathematics, University of Pisa, 56126 Pisa, Italy; 3 Department of Surgical, Medical, Molecular and Critical Area Pathology, University of Pisa, Via Roma 67, 56126 Pisa, Italy; 4 Hepatology Unit and Laboratory of Molecular Genetics and Pathology of Hepatitis Viruses, Reference Center of the Tuscany Region for Chronic Liver Disease and Cancer, Azienda Ospedaliero-Universitaria Pisana, 56126 Pisa, Italy

**Keywords:** NASH, NAFLD, HCV, angiogenesis, BMP-9, HB-EGF, HGF, follistatin

## Abstract

Background: Although liver biopsy remains the gold standard for the diagnosis and the monitoring of liver disease, non-invasive biomarkers have been recently suggested to predict liver disease severity, progression, and response to therapy. We investigated multiple tissue and circulating markers of angiogenesis in predicting the severity of biopsy-validated chronic liver diseases in patients with chronic hepatitis C virus (HCV) and in NAFLD/NASH patients. Methods: We studied samples from forty-six patients with HCV and/or NAFLD who underwent liver biopsy, liver ultrasonography, and liver stiffness measurement. Ishak and Brunt scores were calculated. Expression of selective genes and luminex analyses of 17 different circulating pro-angiogenic factors were performed. Results: The phenotype of NAFLD/NASH or HCV subjects was similar, except for insulin, which was expressed at higher levels in NAFLD/NASH patients. A Mann–Whitney test showed significant differences for the circulating levels of HB-EGF and for follistatin between HCV and NAFLD/NASH patients. In HCV patients, we found an inverse correlation between disease stage and BMP-9 and VEGF-A circulating levels, while in NASH/NAFLD direct correlations between stage and BMP-9 and VEGF-A circulating levels were noted. The K-means algorithm divided HCV and NASH/NAFLD patients in two clusters with significant differences between them. Logistic regression models showed a positive relationship with BMP-9 levels for NASH/NAFLD and with HB-EGF circulating concentrations for HCV. ROC analysis showed for BMP-9 > 1188 pg/mL a worse disease in NASH/NAFLD, whereas for HB-EGF < 61 pg/mL a higher severity of disease in HCV. Conclusion: Our data show that circulating biomarker profiles can identify the severity of chronic liver disease of NAFLD/NASH or HCV origin.

## 1. Introduction

Nonalcoholic fatty liver disease (NAFLD) is defined as the presence of steatosis in >5% of hepatocytes in the absence of significant ongoing or recent alcohol consumption and other known causes of liver disease [1]. NAFLD is increasing in prevalence worldwide; and it has been proposed that this disease be redefined clinically as metabolic-associated fatty liver disease (MAFLD), whereby the pathogenic processes originated from underlying metabolic dysfunction are considered [2]. More than 50% of NAFLD patients progress into nonalcoholic steatohepatitis (NASH), a more severe condition characterized by a certain degree of fibrosis [3]. Although a liver biopsy should be still considered the gold standard for diagnosing and monitoring liver disease, the costs and invasiveness make such a procedure eligible only to selected cases. In the last decade, non-invasive diagnostic biomarkers, the use of transcriptomics, proteomics, and metabolomics to predict liver disease severity, progression, and response to lifestyle changes and pharmacological treatment has been developed [4]. Another area of research consists in characterizing genetic and epigenetic markers, which are useful in evaluating disease progression. These biomarkers taken together with the patient’s clinical history could provide accurate diagnoses, assisting clinicians in early intervention, and improving prognosis.

Angiogenesis is the growth factor-dependent formation of new blood vessels, and it is associated with scar development and sinusoidal remodeling in chronic liver diseases [5]. Multiple factors may initiate angiogenesis in NAFLD, including tissue hypoxia, endothelial dysfunction, hepatic stellate cell activity, and inflammation [6]. In pathological angiogenesis of NAFLD and NASH, there is a strong expression of main pro-angiogenic factors such as vascular endothelial growth factor (VEGF), placenta growth factor (PlGF), Angiopoietin-2 (Ang-2), and platelet derived growth factor (PDGF), which are released by different cell types involved in the progression of chronic liver disease [7]. Additionally, hepatitis C virus (HCV) infection promotes the development of hepatic angiogenesis, and the hepatitis C virus core protein has been described to induce hypoxia-inducible factor 1α-mediated VEGF expression, a main pro-angiogenic factor [8].

Among purinergic receptors, the P2X7 receptor (P2X7R) is involved in angiogenesis modulation in different diseases. P2X7R stimulation promotes mTOR/HIF1α/VEGF signalling, and its blockade fully reverses retinal vascular permeability increase, leads to VEGF accumulation, and increases IL-6 expression in a murine model of diabetic retinopathy [9]. Both P2X7R and P2X4R are upregulated in liver specimens of HCV patients [10].

The potential role of multiple, tissue and circulating, markers of angiogenesis in predicting the severity of biopsy-proven chronic liver diseases in HCV and NAFLD/NASH patients has not been explored so far; the present study has been designed to address this specific issue.

## 2. Materials and Methods

### 2.1. Patients

Forty-six treatment naive patients with chronic hepatitis C and/or NAFLD eligible for liver biopsy because of chronic liver disease associated with fatty liver and/or chronic hepatitis C (CHC) virus infection, without clinical and ultrasound signs of cirrhosis, were consecutively recruited at the Hepatology Unit of the University Hospital in Pisa in the years 2005–2017. We registered anamnestic data, concomitant pharmacologic therapies, and biochemical analyses at the time of the liver biopsy. Forty-eight hours before the liver biopsy (whose indication was posed to clarify and detail the diagnosis) blood samples were drawn from an antecubital vein to determine complete blood count, serum liver enzymes (aspartate aminotransferase [AST], alanine aminotransferase [ALT], gamma-glutamyl transferase [GGT]), liver function tests (albumin, total and direct bilirubin, prothrombin time), lipid profile, and glucose blood levels according to standard laboratory procedures. Additional serum aliquots were collected in the fasting state the day of the biopsy and stored at −20 °C for further determinations, to measure insulin levels (by chemiluminescent immunoassay, LIAISON^®^, DiaSorin S.p.A., Saluggia, Italy), and to perform Luminex analysis.

### 2.2. Liver Stiffness Measurement (LSM)

After overnight fasting, all patients underwent an abdominal ultrasound, and liver stiffness (LS) was measured by FibroScan^®^502 (Echosens, Paris, France) by a single trained physician. Each patient’s LSM was considered adequate if it included at least 10 valid measurements, with a success rate >60% and measurement variability <30% of the median. In this cohort, LSM strongly correlated with biopsy-derived stage scores in both NAFLD (r = 0.60, *p* = 0.003) and in HCV patients (r = 0.76, *p* < 0.0001); therefore, FibroScan values were used to examine the influence of each gene expression on liver fibrosis across different scoring systems.

### 2.3. Liver Biopsy

Sections of formalin-fixed and paraffin-embedded liver specimens were stained with haematoxylin and eosin. The histological examination was performed by an expert liver pathologist who graded inflammation and staged fibrosis according to the Ishak score [11] in CHC liver biopsy and Brunt score for NAFLD [12]. In the Ishak fibrosis scale, a score of 2 is defined as fibrous expansion of most portal areas, with or without short fibrous septa; 3 is defined as fibrous expansion of most portal areas with occasional portal-to-portal bridging; 4 is defined as fibrous expansion of most portal areas with marked bridging (both portal-to-portal and portal-to-central); 5 is defined as incomplete cirrhosis characterized by marked bridging and occasional nodules; and 6 is defined as probable or definite cirrhosis. Brunt criteria include the following parameters: Fibrosis: staged 0 (absent) to 4 (1, perisinusoidal/pericellular fibrosis; 2, periportal fibrosis; 3, bridging fibrosis; 4, cirrhosis); Necroinflamation: graded 0 (absent) to 3 (1, occasional ballooned hepatocytes and no or very mild inflammation; 2, ballooning of hepatocytes and mild to moderate portal inflammation; 3, intra-acinar inflammation and portal inflammation); NASH is defined by the presence of fibrosis (grade 1 or more) or necroinflammation (grade 2 or more).

### 2.4. Gene Expression

Total RNA was extracted from formalin-fixed paraffin-embedded (FFPE) liver sections using RecoverAll Total Nucleic Acid Isolation kit (Ambion, Thermo Fisher Scientific, Waltham, MA, USA). Briefly, 5–10 sections (10 µm thick) were cut from FFPE samples, deparaffinized with xylene, rehydrated in decreasing alcohol series and processed according to manufacturing advice. RNA (1 µg), retrotranscribed with SuperScript Vilo kit (#11754 Thermo Fisher), was analysed using Eco Real-Time system (Illumina Inc., San Diego, CA, USA). Transcripts were evaluated by the following TaqMan Gene Expression Assay (Thermo Fisher): P2X4R, Hs00602442_m1; P2X7R, Hs00175721_m1; NLRP3, Hs00918082_m1; Caspase-1, Hs00354832_m1; AIM2, Hs00915710_m1; IL-2, Hs00174114_m1; GAPDH, Hs02758991_g1. The relative target gene expression, normalized to housekeeping gene GAPDH, is given as 2^−ΔΔCt^, where Ct is the threshold cycle, and referred to their expression in six liver samples collected in patients with liver damage of the ductal plate and minimal/absent necro-inflammation.

### 2.5. Luminex Analysis

Blood was collected in serum tubes (vacutainer tubes; Becton Dickinson Vacutainer System, Rutherford, NJ, USA), temporarily stored for a maximum of 10 min at 4 °C and then centrifuged (10 min, 4000 rpm, 4 °C) to separate serum, which was aliquoted and stored at −80 °C. Fifteen µL of serum from each patient were diluted in assay buffer (1:2 *v:v*, respectively) and then a 25 µL sample of the solution was evaluated by Luminex using the Human Angiogenesis/Growth Factor Panel 1, HAGP1MAG-12K kit purchased from MerckMillipore (Merck KGaA, Darmstadt, Germany).

The samples were loaded into a 96 well-plate supplied by the kit. In each well an equal volume of a premix of 17 luminex beads was added, followed by incubation overnight at 4 °C. The beads were for the following 17 cytokines: Angiopoietin-2 (Ang-2), bone morphogenetic protein (BMP-9), epidermal growth factor (EGF), Endoglin (CD105), Endothelin-1 (ET1), fibroblast growth factor-1 (FGF-1/FGF-acidic), fibroblast growth factor-2 (FGF-2/FGF-basic), Follistatin (FST), Granulocyte colony-stimulating factor (G-CSF), Heparin-binding EGF-like growth factor (HB-EGF), Heparin-binding EGF-like growth factor (HGF), Interleukin-8 (IL-8), Leptin (LEP), Placental growth factor (PLGF), vascular endothelial growth factor-A (VEGF-A), VEGF-C, and VEGF-D. The beads were subsequently washed and incubated with 25 µL of secondary biotinylated detection antibody for 1 h at room temperature, according to the manufacturer’s protocol. The beads were then mixed with 25 µL of streptavidin PE conjugate, washed and resuspended in 100 µL of sheath fluid, and then analyzed by FlexMap3D instrument (MerckMillipore) with xPONENT^®^ software (MerckMillipore) following the manufacturer’s protocols and settings. Assay sensitivities and precision of the analysis are reported in Table 1, including intra-assay and inter-assay % CV’s.

The validated milliplex Human Angiogenesis/Growth Factor Panel 1, HAGP1MAG-12K kit was chosen because, based on the current scientific literature [13], it includes most of the described angiogenic factors (e.g., Angiopoietin, BMP-9, HGF, HB-EGF, FGFs, VEGFs and PLGF) involved in the pathogenesis of liver fibrosis or in liver pathologies.

### 2.6. Statistical Analysis

Based on the result of the Shapiro–Wilk test for normality, continuous data are presented as mean and standard deviation or median and interquartile range. The Chi Square test for the categorical data, and the Mann–Whitney test for continuous variables were utilized to compare two groups.

For each group of patients (HCV and NASH/NAFLD), the main elaboration has been conducted by a k-means clustering, as previously described [14,15], by choosing k equal to 2. Briefly, clustering is the process of separating a group of data points into a small number of k-clusters (in this study k = 2), aimed to assign a cluster to each data point. Data considered to separate each group of subjects were all the available biomarkers. For each group of patients, after the implementation of the clustering algorithm, the differences between the biomarker levels in the two clusters were estimated to identify those that were statistically significant.

All associations were estimated with the Spearman test. The “stage” and “grade” continuous variables were also transformed into logistic variables, where 0 indicates values below the median of the respective set of patients, and 1 value above the median.

Logistic regression models were built, with dependent variable “grade” or “stage”, and predictors those biomarkers that were statistically different between the two clusters.

Finally, receiver operating characteristics (ROC) curves and Y Index were used to estimate optimal cut-points only for the biomarkers previously identified with the regression models.

A *p*-value of 0.05 was the cut-off for significance in all statistical analyses. When necessary, a multiple testing correction was applied. Statistical analysis was performed by IBM SPSS^®^ Statistics package (version 26; IBM, Armonk, NY, USA) for Mac.

## 3. Results

The main clinical characteristics of the study participants are shown in Table 2. The Shapiro–Wilk test showed that age, plasma glucose and serum insulin had normal distribution in HCV patients, while in NAFLD/NASH the normally distributed variables were age, AST, and ALT; all the other parameters had a probability distribution different from the Gaussian. The characteristics of NAFLD/NASH or HCV subjects were similar, except for insulin levels, which were significantly higher in the NAFLD/NASH group. Fibroscan Index values were not significantly different. Some histological characteristics of biopsies are also reported in Table 2.

Tissue expression of the P2X7R-NLRP3 inflammasome components and serum concentration of some circulating biomarkers in the two groups are reported in Table 3. The only two factors showing a normal distribution were CASP1 in HCV and P2X4 in NAFLD/NASH. The Mann–Whitney test corrected for multiple comparisons showed statistically significant differences for the following biomarkers: P2X4, AIM2, IL-2, HB-EGF (lower in HCV), and follistatin (lower in HCV).

**Figure 1:**
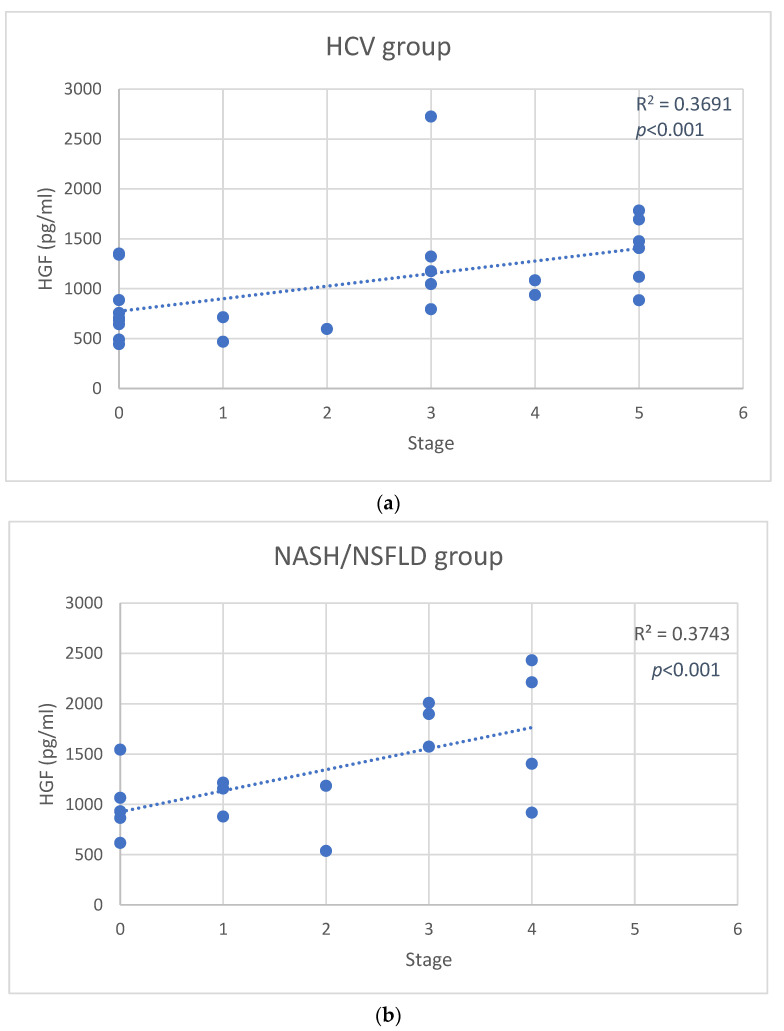
Scatter plot of hepatocyte growth factor (HGF) changes versus stage in the hepatitis C virus (HCV) group (**a**), and in the nonalcoholic steatohepatitis (NASH)/nonalcoholic fatty liver disease (NAFLD) group (**b**). Solid lines represent the linear fit of data.

**Figure 2:**
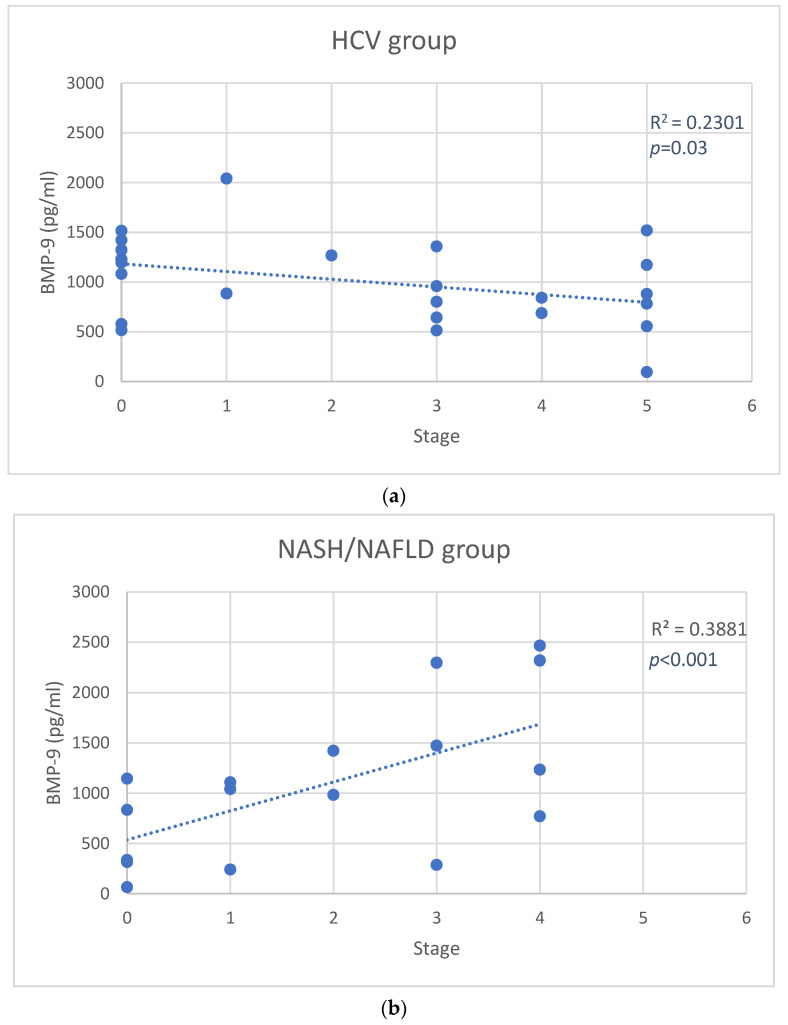
Scatter plot of bone morphogenetic protein-9 (BMP-9) changes versus stage in the hepatitis C virus (HCV) group (**a**), and in the nonalcoholic steatohepatitis (NASH)/nonalcoholic fatty liver disease (NAFLD) group (**b**). Solid lines represent the linear fit of data.

In the HCV group, positive associations between stage and HGF levels (r = 0.61, *p* < 0.001) (Figure 1a), CASP1 (r = 0.61, *p* < 0.001), NRLP3 (r = 0.56, *p* = 0.02) were observed. We also found an inverse correlation between stage and BMP-9 (r = −0.48, *p* = 0.03) (Figure 2a), VEGF-A (r = −0.44, *p* = 0.03), and EGF (r = −0.55, *p* = 0.02) levels. In these patients, grade was negatively related with VEGF-D (r = −0.44, *p* = 0.03), and HB-EGF (r = −0.51, *p* = 0.02) serum levels. Furthermore, HGF concentrations were associated with Fibroscan values (r = 0.56, *p* = 0.02) (Supplementary Figure S1a), but not with ALT levels.

In NASH/NAFLD patients, direct correlations between stage and HGF (r = 0.61, *p* < 0.001) (Figure 1b), BMP-9 (r = 0.62, *p* < 0.001) (Figure 2b), and VEGF-A (r = 0.41, *p* = 0.03) levels emerged, and that, in these subjects, grade was positively associated with VEGF-D (r = 0.48, *p* = 0.03), BMP-9 (r = 0.68, *p* < 0.001), HGF (r = 0.61, *p* < 0.001), and IL-8 (r = 0.58, *p* = 0.02) serum levels. Moreover, BMP-9 levels correlated with Fibroscan (r = 0.57, *p* = 0.02) (Supplementary Figure S1b) but not with ALT levels.

In the HCV group, the k-means algorithm divided the patients into two clusters: cluster 1 including sixteen individuals, and cluster 2 including nine patients. The differences for the following serum levels of investigated factors were statistically significant between clusters, all lower in subjects included into cluster 2: leptin (8050.2 ± 2501.9 vs. 2117.5 ± 988.7 pg/mL, *p* < 0.001), HB-EGF (80.2 ± 61.1 vs. 40.6 ± 23.5 pg/mL, *p* = 0.03), and VEGF-A (560.0 ± 276.3 vs. 292.8 ± 240.6 pg/mL, *p* = 0.02).

Regarding the liver disease staging, the values were higher in individuals belonging to cluster 2 (1.9 ± 2.0 vs. 2.5 ± 2.1, not significant), while the grading levels were comparable (6.6 ± 1.6 vs. 6.7 ± 2.6).

When we applied the clustering algorithm to the NASH/NAFLD group, we observed differences between two clusters (cluster 1: fourteen subjects; cluster 2: seven patients, respectively); all lower serum levels were reported in patients belonging to cluster 1: BMP-9 (911.6 ± 628.4 vs. 2028.6 ± 481.4 pg/mL, *p* = 0.01), leptin (4498.8 ± 2702.4 vs. 11963.9 ± 984.2 pg/mL, *p* <0.001), IL-8 (80.2 ± 63.1 vs. 1168.6 ± 974.8 pg/mL, *p* < 0.001), and HGF (1069.3 ± 336.2 vs. 2040.7 ± 160.4 pg/mL, *p* <0.001).

Regarding the liver disease, both staging and grading levels were lower in individuals belonging to cluster 1 (1.5 ± 1.5 vs. 2.4 ± 1.5, 1.5 ± 0.9 vs. 4.4 ± 2.9, respectively, not significant).

**Figure 3:**
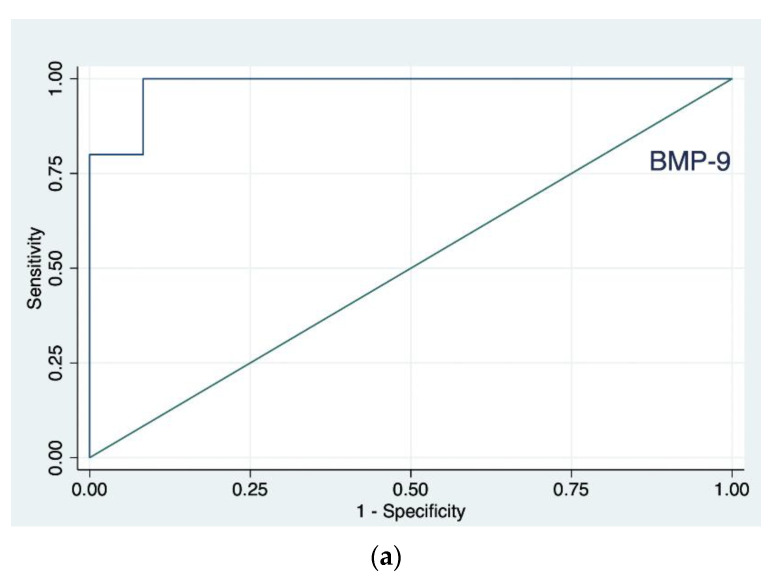

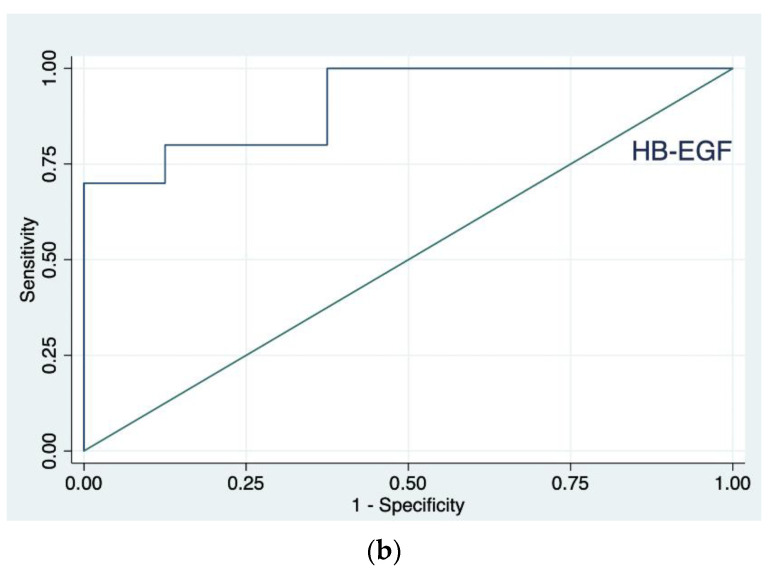
Receiver operating characteristic (ROC) curve for bone morphogenetic protein-9 (BMP-9) values obtained from nonalcoholic steatohepatitis (NASH)/nonalcoholic fatty liver disease (NAFLD) participants, area under the ROC curve (AUC) 0.98 (**a**), and heparin-binding EGF-like growth factor (HB-EGF) values obtained from hepatitis C virus (HCV) participants, area under the ROC curve (AUC) 0.89 (**b**).

Logistic regression models, separately built up in the two groups (NASH/NAFLD and HCV), showed a significant relationship of the grade with the BMP-9 levels, for NASH/NAFLD, and with HB-EGF, for HCV. No significant associations were detected in the logistic models where the response variable was the stage. The ROC analysis (Figure 3) for BMP-9 and HB-EGF, respectively, with estimation of the Youden index, showed for BMP-9 an area under the curve (AUC) of 0.98 and a threshold of 1188 pg/mL (patients correctly classified: 94%), with greater severity in NASH/NAFLD patients with the highest values (Figure 3a). Incidentally, in this group, the only deceased patient had BMP-9 levels of 1144 pg/mL. Instead, for the HB-EGF variable, AUC was 0.89 and the threshold 61 pg/mL (patients correctly classified: 83%), with greater severity in subjects suffering from HCV with lowest levels (Figure 3b). Values in the three deceased patients were equal to 12, 30 and 32 pg/mL.

## 4. Discussion

In the setting of cirrhosis associated with hepatitis B virus (HBV) or with HCV, the risk of development of a hepatocellular carcinoma (HCC) is not trivial. However, in recent years, a progressive increase of HCC in patients with non-viral (mainly metabolic) liver disease has been reported [16]. Several risk scores, including liver stiffness values, age, sex, albumin and, for viral hepatitis, HBV-DNA, have been proposed and used in clinical practice. Their prognostic performance is often insufficient, especially when considering that metabolic liver diseases expose to a high cardiovascular risk [17]. Although liver biopsy is still considered as a gold standard for staging and grading several chronic liver diseases, it is nowadays less utilized than in the past, due to its invasive nature, possibly of sampling error generating misleading results, intra-observer and inter-observer variabilities, high cost, and patient objections. Novel predictive biomarkers of prognosis are urgently needed to guide treatment decision and patient selection, and to better understand and overcome mechanisms of resistance to treatments. Our experimental approach validates some pro-angiogenic factors (e.g., BMP-9 and HB-EGF) as reliable proxies of liver biopsy in a cohort of subjects with metabolic or viral liver disease and a long observational follow up.

Purinergic receptors have been associated with HCV; thus, P2X4R regulates the secretion of micro-RNA containing exosomes by HCV-infected hepatocytes [18], and polymorphisms of genes encoding for inflammasome components, including AIM2, have been correlated to the development of C-hepatitis in Amazonia [19]. Higher expression levels of such molecules are found in blood samples of HCV patients compared to healthy blood donors’ individuals, with a positive association with the disease stage.

Among the significant differences between the NAFLD/NASH and HCV group of patients, it is noteworthy the higher serum levels of the pro-angiogenic factor follistatin in the NAFLD/NASH patients. A recent report by Wu and colleagues [20] found out that elevated circulating follistatin concentrations were associated with an increased risk of type 2 diabetes. Moreover, our data are in accordance with those of Yndestad and collaborators [21], who reported serum levels of follistatin significantly elevated in patients with NAFLD if compared with healthy controls. Furthermore, within a NAFLD group of patients, follistatin was associated with NASH independently from activin A, gender, and age [22].

Based on our data, also HGF levels seem to have an important role in both HCV and NASH/NAFLD diseases because its level is directly related to the stage of the disease (Figure 1), as already described in the scientific literature [23]. Interestingly, the HGF levels correlated with the inflammatory-fibrotic index TE at the Fibroscan, usually used as a marker of hepatic fibrotic/inflammatory damage. However, the role of BMP-9, HB-EGF and VEGF-A serum levels may be different in the two diseases.

BMP-9 is mainly expressed by the liver, and it has been described as a hematopoietic, osteogenic, and chondrogenic factor. Moreover, BMP-9 also modulates glucose and lipid metabolism, angiogenesis, lymphangiogenesis, and liver regeneration [24]. Breitkopf-Heinlein et al. reported the role of BMP-9 as a profibrogenic factor in the liver, but no significant change in BMP-9 expression in patients with hepatitis B virus-associated liver fibrosis was found [25]. Furthermore, preclinical animal models highlighted the importance of BMP-9 in promoting NASH development by directly acting on macrophages [26] or through its pro-inflammatory capacity [27]. Our data show that BMP-9 serum levels are directly correlated with the stage of the disease in NASH/NAFLD patients, and conversely, its concentrations are inversely correlated with the severity of HCV fibrosis. High levels of BMP-9 (>1188 pg/mL) well characterize patients with a greater severity of NASH/NAFLD (ROC analyses AUC 0.98). These results are in accordance with the findings of Li and colleagues, who demonstrated that the serum BMP-9 levels were significantly higher in patients with fibrosis than in healthy subjects, and with significant differences between mild fibrosis and cirrhosis [28]. Additionally of interest is the finding that BMP-9 levels are directly correlated with Fibroscan values, confirming a relationship with the inflammatory/fibrotic damage of the liver. The role of BMP-9, at least in preclinical studies, is still debated. Indeed, a dual role of this protein in hepatic steatosis and fibrosis has been described [29]. While BMP-9 promotes fibrosis by activating hepatic stellate cells (HSCs), it has also been reported that BMP-9 deficiency was associated to a significant hepatic steatosis in a NAFLD mouse model [29]. Nevertheless, our clinical data lean towards a direct correlation between the inflammatory/fibrotic damage and the serum levels of BMP-9.

The origin of circulating biomarkers cannot be restricted to the liver, in particular regarding leptin. Leptin is a peptide hormone mainly secreted by white adipose tissue and it has been described as a proangiogenic factor through the activation of JAK/STAT pathway [30,31]. Leptin concentrations in the peripheral blood of obese people is proportional to the degree of obesity [32], and hyper-leptinemia promotes steatosis with or without NAFLD [33,34]. Indeed, the adipose tissue disfunction in NAFLD is not completely understood but the inflammation of specific depots of white adipose tissue has a key role in NAFLD progression [35,36]. Thus, it is conceivable that in the cluster of NAFLD/NASH group with higher leptin levels, which strongly indicated that a higher BMI, the adipose tissues may be more responsible for circulating leptin.

Another interesting finding of our clinical study was the association of the severity of HCV fibrosis with HB-EGF levels below 61 pg/mL. In general, the HCV patients had lower concentrations of HB-EGF compared to NASH/NAFLD patients (Table 2). Furthermore, after the cluster analysis, a higher stage and grade of HCV fibrosis were predicted by lower serum levels of HB-EGF, with a ROC AUC of 0.89. HB-EGF has been reported to suppress experimental liver fibrosis in mice [37] through the inhibition of HSC activation. Recently, Maretti-Mira and co-workers [38] reported that HB-EGF, the mediator that maintains HSC quiescence, was expressed by liver sinusoidal endothelial cells (LSECs) from both normal and cirrhotic livers, but it was only detected in conditioned medium from normal LSECs in culture.

Our study also supports the concept that pro-angiogenic factors such as vascular endothelial growth factor-A (VEGF-A) are secreted by several liver cell types (e.g., hypoxic hepatocytes, hypoxia-sensitive macrophages) involved in the progression of chronic liver disease [7]. Increased VEGF protein has been described in the liver of rats fed with a choline-depleted amino acid diet with NASH [39]. HSC, portal myofibroblasts, and macrophages, under hypoxic conditions, stimulate angiogenesis by secreting VEGF-A [40]. Interestingly, in our NASH/NAFLD patients, serum VEGF-A levels positively correlate with the fibrosis stage and grade as well, confirming a role of this major angiogenic factor in the pathogenesis of NASH. On the other hand, in HCV patients, VEGF-A levels were inversely correlated with the fibrosis stage and grade, suggesting different hypotheses for the development of the disease.

Our study capitalized on availability of the liver biopsy at baseline, that allowed a proper grading and staging of the disease; a long follow up; up-dated information of the vital status of all subjects. Conversely, the limitations of this study are the small size of the cohort, due to the progressive reduction of clinical indications to liver biopsy, replaced in the last two decades by non-invasive methodologies. Such limited sample size makes difficult to dissect for which variables our set of analyses could perform best in identifying patients. Another important limitation of the present study is the lack of a correlation between gene expression profiles of the liver and the circulating factors in the blood due to the unavailability of residual liver tissue. Indeed, it is likely that serum cytokines did not exclusively come from the liver, since other tissues/cells, such as adipose tissues and peripheral lymphocytes or monocytes, can be key determinants of their circulating levels.

In conclusion, our data reveal novel serum biomarker profiles to identify the severity of chronic liver disease of NAFLD/NASH or HCV origin. These circulating biomarkers included molecules with known pro-angiogenic properties, implying a role for the angiogenesis process in the pathogenesis of chronic liver disease and indicating the target of angiogenesis as new therapeutic approaches for the treatment of liver diseases.

## Figures and Tables

**Table 1:** Assay sensitivities and precision of investigated circulating biomarkers as reported by MerckMillipore HAGP1MAG-12K kit. Intra-assay is generated from the mean of the % CV’s from eight reportable results across two different concentrations of analytes (cytokines) in a single assay. Inter-assay precision is generated from the mean of the % CV’s across two different concentrations of analytes across six different experiments. miniDC, minimum detectable concentrations pg/mL; SD, standard deviation.

Analytes (Cytokines)	miniDC (pg/mL)	miniDC (pg/mL) + 2SD	Intra-Assay %CV	Inter-Assay %CV (*n* = 6 Assays)
Ang-2	3.0	3.0	<10	<20
BMP-9	1.2	1.4	<10	<20
EGF	1.0	1.2	<10	<20
CD105	17.0	22.6	<10	<20
ET1	1.4	1.9	<10	<20
FGF-1/FGF-acidic	4.6	7.4	<10	<20
FGF-2/FGF-basic	8.6	10.5	<10	<20
FST	11.1	13.6	<10	<20
G-CSF	5.4	6.4	<10	<20
HB-EGF	0.4	0.6	<10	<20
HGF	8.5	10.4	<10	<20
IL-8	0.2	0.3	<10	<20
LEP	42.8	51.9	<10	<20
PLGF	0.7	0.9	<10	<20
VEGF-A	8.1	10.6	<10	<20
VEGF-C	7.6	10.7	<10	<20
VEGF-D	1.9	2.4	<10	<20

Angiopoietin-2 (Ang-2), bone morphogenetic protein-9 (BMP-9), epidermal growth factor (EGF), endoglin (CD105), endothelin-1 (ET1), fibroblast growth factor-1 (FGF-1/FGF-acidic), fibroblast growth factor-2 (FGF-2/FGF-basic), follistatin (FST), granulocyte colony-stimulating factor (G-CSF), heparin-binding EGF-like growth factor (HB-EGF), hepatocyte growth factor (HGF), interleukin-8 (IL-8), leptin (LEP), placental growth factor (PLGF), vascular endothelial growth factor-A (VEGF-A), VEGF-C, and VEGF-D.

**Table 2:** Clinical characteristics and biochemical parameters of the study participants.

Variable	NAFLD/NASH *n* = 21	HCV *n* = 25	*p* Value
Age	46.1 (38–53)	43.9 (37–48)	0.26
Men (n, %)	16, 76.2	21, 84.0	0.71
BMI (kg/m^2^)	24.6 (24–29)	24.50 (24–27)	0.58
Glucose (mg/dL)	85.0 (75–115)	84.0 (70–88)	0.14
Insulin (µU/mL)	14.2 (9–22)	7.3 (5–11)	0.03
AST (UI/L)	34.5 (26–49)	44.5 (31–82)	0.08
ALT (UI/L)	52.5 (41–77)	73.5 (46–126)	0.08
GGT (U/L)	92 (36–206)	49 (25–89)	0.09
Total bilirubin (mg/dL)	0.68 (0.57–0.85)	0.95 (0.54–1.29)	0.26
Direct bilirubin (mg/dL)	0.22 (0.18–0.27)	0.22 (0.18–0.37)	0.44
Fibroscan (kPa)	6.2 (5.2–11.9)	7.5 (6.1–12.4)	0.14
Platelets (1000/μL)	219 (168–267)	207 (170–238)	0.55
Triglycerides (mg/dL)	108 (90–220)	84 (69–128)	0.16
HDL-cholesterol (mg/dL)	45 (40–50)	41 (29–46)	0.24
LDL-cholesterol (mg/dL)	124 (99–178)	96.5 (66–135)	0.19
Albumin (mg/dL)	4.7 (4.2–4.8)	4.6 (4.4–4.7)	0.64
INR	1.06 (1.00–1.12)	1.03 (1.01–1.08)	0.62
Only steatosis (grading 0; n)	0	0	==
Ishak staging score > 1 (n)	==	14	==
Ishak staging score 6	==	0	==
Brunt staging score 4	4	0	==

Data are reported as median (range). Statistically significant differences are in bold. Alanine aminotransferase (ALT), aspartate aminotransferase (AST), body mass index (BMI), gamma-glutamyl transferase (GGT), hepatitis C virus (HCV), high-density lipoprotein (HDL), international normalized ratio (INR), low-density lipoprotein (LDL), nonalcoholic fatty liver disease (NAFLD), nonalcoholic steatohepatitis (NASH).

**Table 3:** Tissue and circulating biomarkers significantly different between HCV and NAFLD/NASH patients.

Variable	NAFLD/NASH	HCV	*p* Value
Tissue-Based Biomarkers			
**P2X4 (T/R)**	**0.5 (0.3–0.7)**	**1.3 (0.7–2.6)**	**0.02**
P2X7 (T/R)	0.3 (0.3–1.0)	1.2 (0.8–2.2)	0.12
NRLP3 (T/R)	0.6 (0.5–1.4)	1.0 (0.6–2.2)	0.07
**AIM2 (T/R)**	**0.1 (0.0–0.3)**	**0.5 (0.2–1.0)**	**0.04**
CASP1 (T/R)	0.4 (0.4–0.6)	0.9 (0.5–1.0)	0.07
**IL-2 (T/R)**	**0.1 (0.0–0.7)**	**0.9 (0.6–1.3)**	**0.03**
**Circulating Biomarkers**			
IL-8 (pg/mL)	89.9 (46.1–204.3)	24.7 (13.1–52.3)	0.40
**Follistatin (pg/mL)**	**310.2 (240.1–420.6)**	**197.4 (156.0–228.3)**	**0.01**
Angiopoietin (pg/mL)	545.9 (430.1–1040.0)	703.1 (376.6–1160.7)	0.48
G-CSF (pg/mL)	55.6 (36.4–66.6)	73.4 (47.4–106.1)	0.08
BMP-9 (pg/mL)	1041.5 (408.9–1374.7)	958.1 (686.8–1320.3)	0.70
Endoglin (pg/mL)	2364.2 (1629.6–2744.6)	2362.0 (1854.7–2862.1)	0.78
Leptin (pg/mL)	6772.6 (3364.7–10,566.6)	2908.4 (1603.8–7478.4)	0.56
HGF (pg/mL)	1184.9 (889.0–1567.2)	937.3 (707.5–1339.8)	0.13
EGF (pg/mL)	515.5 (378.5–545.0)	514.2 (366.0–657.4)	0.41
**HB-EGF (pg/mL)**	**91.0 (44.9–107.5)**	**48.8 (27.1–62.0)**	**0.02**
FGF-1 (pg/mL)	1.6 (0.3–3.2)	2.7 (1.1–4.3)	0.86
FGF-2 (pg/mL)	74.5 (51.4–85.5)	99.1 (50.0–125.2)	0.12
VEGF-A (pg/mL)	435.6 (279.2–729.3)	299.9 (193.3–385.8)	0.08
VEGF-C (pg/mL)	2668.6 (1852.5–2904.8)	2244.7 (1247.0–3071.7)	0.81
VEGF-D (pg/mL)	892.7 (412.7–1349.0)	968.5 (580.8–1964.7)	0.96
PLGF (pg/mL)	3.4 (1.8–4.9)	5.1 (3.0–7.3)	0.85

Data are reported as median (interquartile range). Statistically significant differences are in bold. Absent in melanoma 2 (AIM2), bone morphogenetic protein-9 (BMP-9), caspase-1 (CASP1), epidermal growth factor (EGF), endothelin-1 (ET1), fibroblast growth factor-1 (FGF-1/FGF-acidic), fibroblast growth factor-2 (FGF-2/FGF-basic), granulocyte colony-stimulating factor (G-CSF), heparin-binding EGF-like growth factor (HB-EGF), hepatitis C virus (HCV), hepatocyte growth factor (HGF), interleukin-2 (IL-2), interleukin-8 (IL-8), nonalcoholic fatty liver disease (NAFLD), nonalcoholic steatohepatitis (NASH), Nucleotide-binding oligomerization domain, Leucine rich Repeat and Pyrin domain containing 3 (NRLP3), P2X purinoceptor 4 (P2X4), P2X purinoceptor 7 (P2X7), placental growth factor (PLGF), vascular endothelial growth factor-A (VEGF-A), VEGF-C, and VEGF-D.

## Data Availability

The data are available at the Department of Clinical and Experimental Medicine (Guido Bocci).

## Supplementary Materials

The following supporting information can be downloaded at: https://www.mdpi.com/article/10.3390/jcm11205985/s1. Figure S1. Scatter plot of HGF changes versus Fibroscan in HCV group (a), and of BMP-9 changes versus Fibroscan in NASH/NAFLD group (b). Solid lines represent the linear fit of data.

## Abbreviations

AIM2	absent in melanoma 2
ALT	alanine aminotransferase
Ang-2	angiopoietin-2
AST	aspartate aminotransferase
AUC	area under the curve
BMI	Body Mass Index
BMP-9	bone morphogenetic protein-9
CAP	controlled attenuation parameter
CASP1	caspase-1
CD105	endoglin
EGF	epidermal growth factor,
ET1	endothelin-1
FGF-1	fibroblast growth factor-1
FGF-2	fibroblast growth factor-2
FST	follistatin
G-CSF	granulocyte colony-stimulating factor
GGT	gamma-glutamyl transferase
HB-EGF	heparin-binding EGF-like growth factor
HCC	hepatocellular carcinoma
HCV	hepatitis C virus
HDL	High-density lipoprotein
HGF	hepatocyte growth factor,
IL-2	interleukin-2
IL-8	interleukin-8
INR	International Normalized Ratio
LDL	Low-density lipoprotein
LEP	leptin
LSM	Liver stiffness measurement
NAFLD	Nonalcoholic fatty liver disease
NASH	nonalcoholic steatohepatitis
NRLP3	Nucleotide-binding oligomerization domain, Leucine rich Repeat and Pyrin domain containing 3
P2X4R	P2X purinoceptor 4
P2X7R	P2X purinoceptor 7
PLGF	placental growth factor
ROC	receiver operating characteristics
T/R	target-reference ratio
VEGF-A	vascular endothelial growth factor-A
VEGF-C	vascular endothelial growth factor-C
VEGF-D	vascular endothelial growth factor-D
