# Supplementary material for: Predictive Power of Tissue and Circulating Biomarkers for the Severity of Biopsy-Validated Chronic Liver Diseases
Source: J Clin Med. 2022 Oct 11;11(20):5985. doi: 10.3390/jcm11205985 (PMC9604565; doi:10.3390/jcm11205985)
Supplement: Supplementary file 1 [file jcm-11-05985-s001.zip › supplementary figure S1.pdf]

a

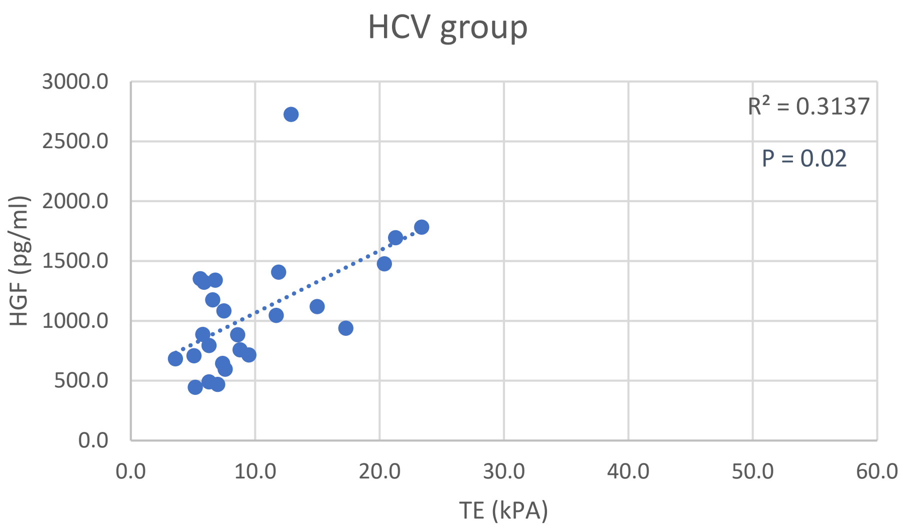

b

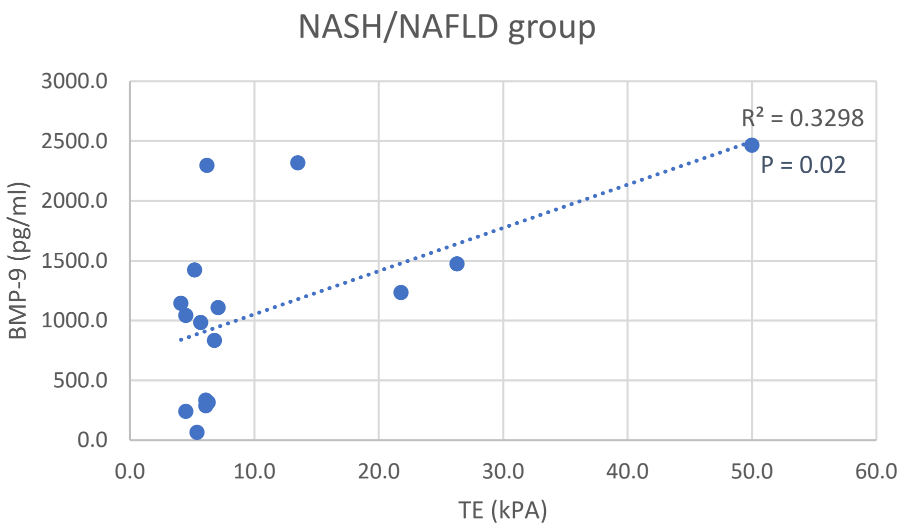

Supplementary Figure S1. Scatter plot of HGF changes versus Fibroscan in HCV group (**a**), and of BMP-9 changes versus Fibroscan in NASH/NAFLD group (**b**). Solid lines represent the linear fit of data.
